# Untargeted analysis of the airway proteomes of children with respiratory infections using mass spectrometry based proteomics

**DOI:** 10.1038/s41598-018-32072-3

**Published:** 2018-09-14

**Authors:** Charles J. Sande, Martin Mutunga, Jacqueline Muteti, James A. Berkley, D. James Nokes, James Njunge

**Affiliations:** 1 KEMRI-Wellcome Trust Research Programme, Kilifi, Kenya; 2 Centre for Tropical Medicine and Global Health, Nuffield Department of Medicine, University of Oxford, Oxford, United Kingdom; 3 School of Life Sciences and Zeeman Institute (SBIDER), University of Warwick, Coventry, United Kingdom

## Abstract

The upper airway – which consists mainly of the naso- and oro-pharynx - is the first point of contact between the respiratory system and microbial organisms that are ubiquitous in the environment. It has evolved highly specialised functions to address these constant threats whilst facilitating seamless respiratory exchange with the lower respiratory tract. Dysregulation of its critical homeostatic and defence functions can lead to ingress of pathogens into the lower respiratory tract, potentially leading to serious illness. Systems-wide proteomic tools may facilitate a better understanding of mechanisms in the upper airways in health and disease. In this study, we aimed to develop a mass spectrometry based proteomics method for characterizing the upper airways proteome. Naso- and oropharyngeal swab samples used in all our experiments had been eluted in the Universal Transport Media (UTM) containing significantly high levels of bovine serum albumin. Our proteomic experiments tested the optimal approach to characterize airway proteome on swab samples eluted in UTM based on the number of proteins identified without BSA depletion (Total proteome: Protocol A) and with its depletion using a commercial kit; Allprep, Qiagen (cellular proteome: Protocol B, C*i*, and C*ii*). Observations and lessons drawn from protocol A, fed into the design and implementation of protocol B, and from B to protocol C*i* and finally C*ii*. Label free proteome quantification was used in Protocol A (n = 6) and B (n = 4) while commercial TMT 10plex reagents were used for protocols C*i* and *ii* (n = 83). Protocols C*i* and *ii* were carried out under similar conditions except for the elution gradient: 3 h and 6 h respectively. Swab samples tested in this study were from infants and children with and without upper respiratory tract infections from Kilifi County Hospital on the Kenyan Coast. Protocol A had the least number of proteins identified (215) while B produced the highest number of protein identifications (2396). When Protocol B was modified through sample multiplexing with TMT to enable higher throughput (Protocol C*i*), the number of protein identified reduced to 1432. Modification of protocol C*i* by increasing the peptide elution time generated Protocol C*ii* that substantially increased the number of proteins identified to 1875. The coefficient of variation among the TMT runs in Protocol C*ii* was <20%. There was substantial overlap in the identity of proteins using the four protocols. Our method was were able to identify marker proteins characteristically expressed in the upper airway. We found high expression levels of signature nasopharyngeal and oral proteins, including BPIFA1/2 and AMY1A, as well as a high abundance of proteins related to innate and adaptive immune function in the upper airway. We have developed a sensitive systems-level proteomic assay for the systematic quantification of naso-oro-pharyngeal proteins. The assay will advance mechanistic studies of respiratory pathology, by providing an untargeted and hypothesis-free approach of examining the airway proteome.

## Introduction

The human naso-oro-pharynx constitutes the first line of mechanical and immunological defence against infectious pathogens and particulate pollutants in the air. Pathogens and commensal microorganisms constantly interact with the cells of the upper airway^1–3^ and in order to maintain homeostasis, humans have evolved complex and dynamic defence mechanisms that maintain a functional mucosal barrier^4^. This barrier regulates microbial colonisation and maintains a potent immunological defence against pathogenic micro-organisms^5^. The effector functions that underpin this regulatory role are a product of the airway proteome, which is derived mainly from epithelial cells as well as innate and adaptive immune cells^6–8^. As part of these regulatory mechanisms, incipient antigens are initially expelled by non-immunological mechanisms such as mucus entrapment, enzymatic degradation and through mechanical egress via mucociliary action^9–12^. Endosomal degradation by phagocytes such as neutrophils and macrophages as well as other non-specific immunological mechanisms such as complement activation may be employed for antigens that persist. The final layer of immunological defence in the upper airway consists of adaptive immune responses – such as antibody responses - that are specifically tailored to specific pathogens^9^. In spite of its crucial role in the maintenance of airway health, the proteome of naso-oro-pharynx remains poorly studied, particularly in infants and children with serious respiratory infections. The naso-oro-pharynx and its secretions provide an excellent resource for identifying potential biomarkers of disease and could contribute substantially to the understanding of mechanisms of airway diseases.

Mass spectrometry-based proteomics combined with computational analysis has become a powerful tool for large-scale systematic investigation of biological processes in an untargeted and hypothesis-free approach^13^. Proteomics has been employed to profile the nasal cavity and mucous proteome in general^14–19^, as well as investigate airway diseases such as allergic rhinitis^14,20–25^, chronic rhinosinusitis^26–28^, and cystic fibrosis^29,30^. It is additionally notable that mass spectrometry platforms used to investigate protein expression in these studies may have previously lacked sufficient sensitivity necessary for extensive proteome coverage that would be helpful in elucidating important biomarkers of mechanisms or prognosis. In this study, we developed a high-throughput swab proteomics workflow that can be used to investigate the airway proteomes of infants and children with serious respiratory illnesses especially from hospitals and field samples.

## Materials and Methods

### Study population and sampling procedures

Naso-oro-pharyngeal samples were collected using nasal and oropharyngeal (NPOP) swabs (Copan Diagnostics, USA) and were eluted in 3 ml of Universal Transport Media (UTM, Copan Diagnostics, USA) that had been supplemented with 500ul of a protein/DNA/RNA preservative, Allprotect (Qiagen, Germany). Upon collection, samples were stored at −80 °C. The study population consisted a total of 93 infants and young children admitted to Kilifi County hospital, in coastal Kenya, with respiratory infections and control infants who were sampled from home and did not exhibit any respiratory symptoms (Protocol A: n = 6, Protocol B: n = 4, Protocol C: n = 83). Diagnosis of respiratory infections was done using a multiplex PCR that was designed to detect 15 respiratory pathogens: respiratory syncytial virus (RSV - A & B), rhinovirus, parainfluenza virus (1, 2, 3 & 4) adenovirus, influenza (A, B & C), coronavirus (OC43 & e229), human metapneumovirus and Mycoplasma pneumoniae. Written informed consent was sought from the parents or legal guardians of the recruits prior to sampling while ethical clearance for the conduct of this study was provided by the Kenya Medical Research Institute (KEMRI) Scientific and Ethical Review Committee (SERU). All methods were performed in accordance with good clinical laboratory practice (GCLP) guidelines.

### Overview of protocol development for analysis of the upper-airway proteome

**Figure 1:**
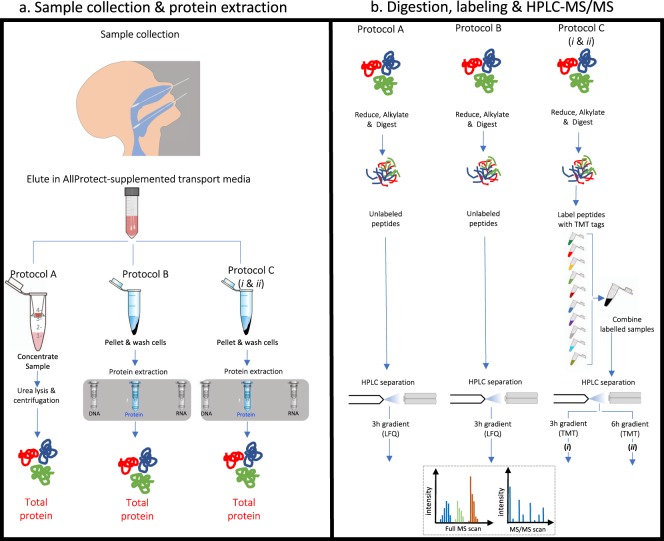
A schematic overview of the naso-oro-pharyngeal proteomics experiments: Naso- and oro-pharyngeal swabs were collected from infants and eluted in universal transport media supplemented with a protein/DNA/RNA preservative. In one set of samples (protocol A) cells in UTM were lysed with urea, reduced, alkylated and digested with trypsin. Peptides were then separated by HPLC and eluted in a 3 h gradient prior to MS analysis. In another set of samples (protocols B & C) cells in UTM were pelleted by centrifugation, lysed and proteins extracted using a commercial extraction kit. Proteins were then reduced, alkylated and digested with trypsin. In protocol B, peptides were separated by HPLC and eluted on a 3 h gradient. In protocol C peptides were labelled using TMT isobaric mass tags and pooled together prior to HPLC separation. In protocol C*i*, proteins were eluted on a 3 h gradient, while in protocol C*ii*, proteins were eluted on a 6 h gradient prior to MS analysis.

Our proteomic experiments tested the optimal approach to characterize airway proteome on swab samples eluted in UTM based on the number of proteins identified. Protocols were developed and tested in a stepwise sequence, with observations and lessons drawn from one protocol, feeding into the design and implementation of the next. The first protocol that was tested (A) was based on six randomly selected patient samples which were lysed with urea, peptides generated using a standard workflow and analysed by label free quantification. In the second protocol (B) nasal samples from four randomly selected children were selected and cells were obtained by centrifugation. The cells were then lysed, total protein extracted from the lysate using a commercial kit (Allprep, Qiagen), peptides generated and analysed by label free quantification. In the third and fourth protocols (C*i* and C*ii*) samples were selected from 83 children. In protocol C, proteins and peptides were prepared in the same manner as protocol B, after which peptides were labelled with isobaric mass tags (TMT10plex). Protocols C*i* and *ii* were carried out under similar conditions except for the elution gradient: 3 h and 6 h respectively. A schematic outline of all the protocols is shown in Fig. 1 while a detailed description of the individual protocols is outlined in supplementary methods.

### Mass spectrometry analysis

Peptides (8 μl) were loaded using a Dionex Ultimate 3000 nano-flow ultra-high-pressure liquid chromatography system (Thermo Scientific, USA) on to a 75 µm × 2 cm C18 trap column (Thermo Scientific, USA) and separated on a 75 µm × 50 cm C18 reverse-phase analytical column (Thermo Scientific) at heated at 40 °C. For LFQ protein quantification; elution was carried out with mobile phase B (80% acetonitrile with 0.1% formic acid) gradient (4 to 30%) over 180 min at a flow rate of 0.25 μl/min. For TMT protein quantification, two gradients were employed: in Protocol C_*i*,_ the gradient was similar to that used for LFQ protein quantification, while the gradient employed in protocol C_*ii*_ was modified to elute peptides for 310 min. Each LC run was finished by washout with 98% B for 10 min and re-equilibration in 2% B for 30 min. Five blanks of 40 min each were run on the column between each injection comprising of two wash cycles with 90% B and an equilibration phase of 15 min to avoid sample carryover. Peptides were measured using a Q Exactive Orbitrap mass spectrometer (Thermo Scientific, USA) coupled to the chromatography system via a nano-electrospray ion source (Thermo Scientific). On the Q Exactive, the ms^1 settings for peptides were: Resolution, 70000; AGC target, 3e6; maximum IT, 120 ms; scan range, 400–1800 m/z; while the ms^2 settings for fragmentation spectra of peptides were: Resolution, 17000 (35000 for labelled peptides); AGC target, 5e4; maximum IT, 120 ms; isolation window, 1.6 m/z. MS data were acquired by data dependent acquisition where the top 12 (15 for labelled peptides) most intense precursor ions in positive mode were selected for ms^2 Higher-energy C-trap dissociation fragmentation which were subsequently excluded for the next 45 s following fragmentation event. Charge exclusion was set to ignore peptide spectrum matches that were unassigned, singly charged, and those with ≥+8 charges.

### Data processing

Raw mass spectrometer files were analysed by MaxQuant software version 1.6.0.1^31^ by searching against the human Uniprot FASTA database (downloaded February 2014) using the Andromeda search engine^32^.

#### For LFQ protein quantification

Cysteine carbamidomethylation was set as a fixed modification and N-terminal acetylation and methionine oxidations as variable modifications. The false discovery rate (FDR) was set to 0.01 for both proteins and peptide-spectrum matches and was determined by searching a FASTA protein database comprising target and reversed target sequeces (decoy) derived from the organism being studied, by switching the amino-carboxyl orientation of a protein’s amino acids to generate sequences that do not exist in nature^33^. Enzyme specificity was set as C-terminal to arginine and lysine with trypsin as the protease. A maximum of two missed cleavages were allowed in the database search. Peptide identification was performed with an allowed initial precursor mass deviation of up to 7 ppm and an allowed fragment mass deviation of up to 20 ppm. A minimum peptide length of 7 amino acids and a maximum peptide mass of 4600 Da was allowed for the searches. The label free quantification (LFQ) algorithm in MaxQuant was used to obtain quantification intensity values.

#### For labelled protein quantification

The 10plex TMT was specified under isobaric labels for reporter ion MS^2 and reporter mass tolerance was set at 0.01 Da. Carbamidomethylation(C) and TMT-10plex labeled N-terminus and lysine were set as a fixed modification while Oxidation (M) and Acetylation (Protein N-term) as variable modifications with both types of modifications being used for protein quantification. The 10plex reporter ion intensity matrix for the study participants was extracted from the Maxquant proteingroup matrix file and batch corrected using an in house platform.

## Results

### Assay-performance

**Figure 2:**
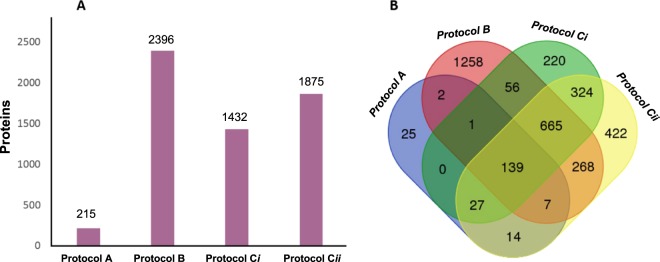
The number of proteins identified by the four methods evaluated are shown in (**A**) The total number of protein identifications in protcols A,B,Ci & Cii were 215, 2396, 1432 and 1875 respectively. The overlap in the identity of prteins identified by the four methods – i.e. the proteins that were common in the different experiments are shown in panel (**B**).

The performance of the four test protocols was based on the number of proteins identified in each protocol as well as by the presence of marker proteins that are known to be expressed by cells of the upper respiratory tract. Of the four protocols, Protocol B – in which proteins were extracted from the patient samples using a commercial Protein/DNA/RNA extraction kit, followed by label free quantification – resulted in the highest number of protein identifications (Fig. 2A) while protocol A – where samples were lysed with urea and then reduced, alkylated and digested, followed by label-free quantification – resulted in the lowest number of protein identifications. Protocol C, which comprised two sub-protocols, distinguished by the elution gradient, resulted in an intermediate number of identifications, although, Protocol C*ii* – where proteins were extracted using the commercial extraction kit, peptides labelled with TMT tags and subsequently eluted on a 310-minute gradient – yielded a substantial increase in protein identifications over protocol C*i*. Of the three protocols that yielded the highest number of protein identifications (protocols B, C*i* & C*ii*) there was a substantial overlap in the identity of proteins detected by the three methods as shown in Fig. 2B.

**Figure 3:**
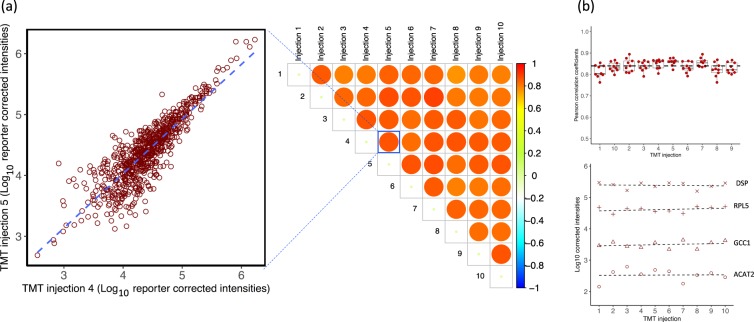
Correlation between the proteomes of the pooled control sample present in different batches (injections) was analysed using pearson correlation analysis. (**a**) pairwise correlation coefficents are plotted on the distance matrix shown on the right - ranging from zero (no correlation; blue) to one (perfect correlation; red). On the left, an example of these correlations between batch 4 and batch 5 is shown. (**b**) Top panel - the pairwise correlation coefficients of the control sample in each batch on the x-axis and the other 9 batches is shown. The dotted line denotes the median of the pairwise correlation coeffcients (0.85). Bottom panel – the inter-batch expression levels of five proteins in the pooled control (ACAT2, GCC1, RPL5 and DSP) whose median expression levels spanned the entire dynamic range of protocol C*ii* is shown. The expression levels of these proteins remained relatively stable, irrespective of the batch in which they were analysed.

Further analysis of assay stability and reproducibility was restricted to samples from protocol C*ii*, which was deemed to be the most practical for routine application due to its capacity for multiplexing and superior performance in protein identification compared to protocol C*i*. The inter-batch correlation of the total proteome of the pooled control sample was carried out by calculating pairwise Pearson’s correlation coefficients between batches. As shown in Fig. 3a, there was a high degree of correlation between batches, with a median Pearson’s R value of 0.85 (Fig. 3b). A random selection of 4 proteins (ACAT2, GCC1, RPL5 & DSP) whose median expression levels were spread out across the entire dynamic range of protocol C*ii*, showed that the expression levels of these proteins remained relatively stable across all experimental batches (Fig. 3c).

### Cell-specific marker protein analysis

**Figure 4:**
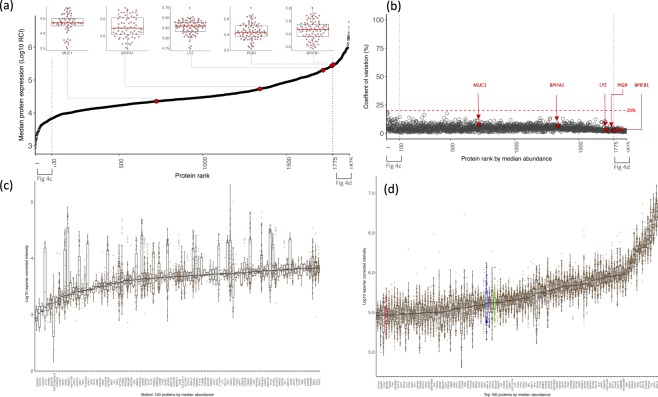
(**a**) All proteins in the naso-oro-pahryngeal proteomes evaluated in protocol C*ii* are ranked in the order of median expression and marker proteins (MUC1, BPIFA1, LYZ, PIGR and BPIFB1) highlighted in red. Their relative distributions are shown inset. (**b**) Coeffients of variation (CV) were calculated for all proteins in the control sample that was common to the 10 experimental batches of protocol Cii. Marker proteins are highlighted in red. The coefficients of variation of all samples was below 20%. The relative distributions of bottom 100 proteins by median abundance are shown in (**c**) while the top 100 proteins by median abundance are shown in (**d**). Highlighted in red, blue and green respectively are the distributions of the naso-oro-pharyngeal marker proteins, BPIFA1, MUC5 and AMY1A which were among the top 100 most abundant proteins by median expression.

In order to further characterise the proteomes identified in protocol C*ii*, the expression of signature proteins that are typically expressed by upper-airway cells was assessed. High expression levels of the signature nasopharyngeal proteins BPIFA1 and BPIFA2 was noted (Fig. 4a). The coefficients of variation for these proteins as well as other proteins in the pooled control was less than 20% (Fig. 4b). In addition, mucins -MUC5 and MUC1 – major components of the mucinous secretions that ubiquitously coat mucosal surfaces of the respiratory tract, were expressed at high levels in the nasopharyngeal secretions of all infants in the study (Fig. 4a & 4d). The two most highly expressed proteins in the naso-oro-pharyngeal proteome were the signature mucosal epithelia keratins, keratin 13 (KRT13) and keratin 4 (KRT4) – Fig. 4d. In addition to these nasopharyngeal marker proteins, we also noted high expression levels of definitive oral/salivary proteins such as salivary amylase in all samples that were analysed – Fig. 4d. Other proteins such as the polymeric immunoglobulin receptor (PIGR), a protein that mediates active trans-cytosis of secretory immunoglobulins onto the luminal surface of the respiratory tract – were expressed at high levels in all patient samples analysed – Fig. 4d.

### Analysis of the proteome of non-epithelial cells

**Figure 5:**
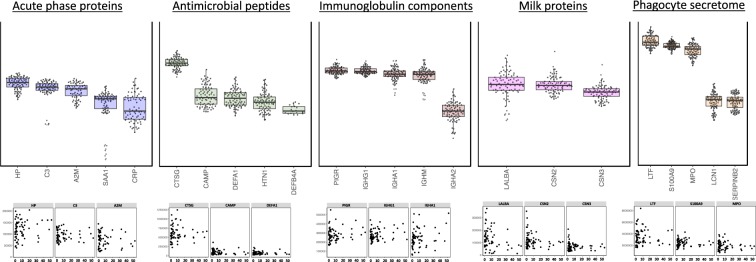
Naso-oropharyngeal proteins were stratified into five functional categories and the relative expression distributions of representative proteins within each category plotted. At the bottom of each category, the age (in months) distribution of the top three most highly expressed proteins in each category is plotted. With the exception of milk proteins, which were highly abundant in the first six months of life, no other proteins appear to vary systematically with age.

In addition to proteins whose origins could be attributed to resident epithelial cells, we noted the presence of proteins secreted by non-epithelial cells. We evaluated the expression levels of different sets of proteins stratified by their putative origins, including acute phase proteins (APP), antimicrobial peptides (AMP), immunoglobulin proteins, milk proteins and proteins associated with the effector functions of phagocytic cells. There was a high degree of heterogeneity in the expression level of acute phase proteins, with the median expression level of haptoglobin (HP) being the highest in this category while C-reactive protein (CRP) had the lowest median expression level among the APPs analysed - Fig. 5a (first panel). A similarly diverse pattern of expression was also observed among antimicrobial peptides, with Cathepsin G (CTSG), being the most abundant AMP while the alpha defensin, DEFA1, was expressed at much lower levels – Fig. 5a (second panel). In addition to APPs and AMPs, extremely high expression levels of immunoglobulin heavy chains (alpha, gamma and mu) were observed in the naso-oro-pharyngeal secretions of all the children in the study, although, the IgA heavy chain, IGAH2, was expressed at much lower levels compared to the other immunoglobulin subclass heavy chains – Fig. 5a (third panel). The final set of immune-related proteins was associated with the effector functions of phagocytic cells such as neutrophils and macrophages – Fig. 5a (fifth panel). Three proteins in this category, lactotransferrin (LTF), S100 Calcium and zinc binding protein S100A9, a component of calprotectin (S100A8/9) and myeloperoxidase (MPO) were among the most highly expressed proteins in the entire naso-oro-pharyngeal proteomes – Fig. 4d - with their respective median expression levels being exceeded only by the cytoskeletal and microfibrillar mucosal keratins (KRT4 and KRT13) and beta-actin (ACTB). In addition to these host proteins, we also identified high levels of non-host proteins in the respiratory tract. The most highly expressed of these were three milk proteins: lactalbumin alpha (LALBA), beta casein (CSN2) and kappa casein (CSN3) – Fig. 5a (fourth panel). Validation of whether these milk proteins were of human or bovine origin was not carried out. To determine the age correlates of these proteins, the respective expression levels of the top three proteins in each functional category was plotted against the age of the study participants – Fig. 5b. With the exception of milk proteins, no systematic changes in protein expression level could be associated with increasing age. In the case of the milk proteins, all three appeared to be most abundant in the first 6–12 months of life, after which a precipitous decline in later years was observed – Fig. 5b (fourth panel). Finally, we investigated the expression of selected T-cell associated pro-inflammatory proteins. Interferons (IFN-α/β/γ), T-cell phenotypic markers (CD3/4/8) or their associated effector proteins (IL4/5/13/17/10, Granzyme) were not detected in the naso-oro-pharyngeal proteomes of any of the study subjects.

### Cluster analysis of protein expression between children with respiratory infections and healthy controls

Using the data from protocol Cii, we undertook ordination analysis to visualize whether the proteome of children with respiratory infections of varying severities and healthy controls sampled from home varied. Using nonmetric multidimensional scaling, we found that the airway proteomes of children admitted to hospital with severe respiratory infections and those with mild infections that did not warrant hospitalisation, largely overlapped (supplementary Figure 1). However, the proteomes of healthy control children who were sampled from home, clustered separately from those with respiratory infections.

## Discussion

We describe the development of a comprehensive method of analysing the naso-oro-pharyngeal proteome of infants and young children with respiratory illnesses. Of the four protein identification methods reported, the highest number of protein identifications was achieved using protocol B, a method in which proteins in naso-oro-pharyngeal samples were extracted using a commercial extraction kit, followed by label-free quantification. However, unlike the methods described in protocol C, this method is limited by the fact that only one sample can be analysed at a time, limiting its practicality for high-throughput applications. In protocols C*i* and C*ii*, peptides from different patient samples were labelled with TMT10plex reagents, allowing for sample multiplexing prior to MS analysis, followed in-silico de-multiplexing after analysis. This analytical design not only increases sample throughput, but concurrently limits the inter-sample variance that is inherent to single-sample analysis. For this reason, we limited further analysis of assay sensitivity, stability and reproducibility to protocols C*i* & C*ii*. We found high levels of correlation between control samples run on different assay batches as well as limited inter-batch variance, suggesting that the methods developed were sufficiently robust for the quantitative analysis of the upper-airway proteome.

In order to increase the sensitivity of protein identification, we experimented on the effect of different elution gradients on protein identification. In the initial set of experiments in protocol C*i*, peptides were eluted for 180 minutes, resulting 1,432 protein identifications, however when the elution time was increased to 310 minutes - (protocol C*ii*) – a further 443 proteins were identified, bringing the total number of proteins identified in protocol C*ii* to 1,875. Compared to previous mass-spectrometry-based proteomics studies of the upper airway, the number of proteins identified in protocol C*ii*, vastly exceeded the number of proteins reported in previously published reports^14,20,22,23,25,26,28,29^.

Further characterisation the proteomes identified in protocol C*ii*, was done by evaluating the expression levels of marker proteins of resident naso-oro-pharyngeal epithelial cells. In-line with recent studies of the upper airway transcriptome^34^ we found consistently high expression of the signature upper-airway proteins BPIFA1 and BPIFA2. These proteins have a unique expression profile, which is restricted to the trachea, nasopharyngeal epithelia and salivary gland^35^ and are therefore considered to be the signature proteins of the upper airway. In addition to these definitive upper-airway proteins, we found a consistently high level of expression of mucins (MUC1 & 5) - the main component of respiratory tract mucus – and mucosal-associated keratins, KRT4 and KRT13. These keratins were the most abundantly expressed of all proteins detected in the naso-oropharyngeal proteome. KRT4 and KRT13 are both differentiation keratins of the oral mucosa^36^ and therefore the high levels at which they were detected is consistent with the anatomical locations that were targeted for sampling. In addition to these upper-airway differentiation proteins, we found high expression levels of a number of antibody related proteins, including the polymeric immunoglobulin receptor, PIGR. PIGR is a type I, membrane-spanning antibody Fc receptor that is expressed at high levels by mucosal epithelial cells and facilitates the active transcytosis of secretory immunoglobulins^37^. The expression of PIGR was linked to the equally high expression of the immunoglobulin heavy chains alpha(IgA), mu(IgM) and gamma(IgG), an association that aligns with the known biological function of PIGR and likely indicates its role in maintaining high levels of these antibodies on the mucosal surface.

We also examined the secretome of immune cells in the airway. We found a large number of proteins that are associated with innate immunity; which in most cases, were expressed at very high levels. For example, the expression levels of the neutrophil antimicrobial effector proteins LTF, S100A9 and MPO, were not only among the highest of the entire naso-oropharyngeal proteome, but they were also present in the upper-airway proteome of each child in this study. The observed overabundance of proteins these proteins, as well others also related to antimicrobial immunity like CTSG, CAMP,DEFA1/2 and HTN1 is most likely related to the need to prevent bacterial overgrowth and maintain homeostatic balance in the upper respiratory tract. Previous studies have provided compelling evidence of the functional role of proteins such as LTF, CAMP, CTSG and others in limiting bacterial replication^38,39^ and the presence of these proteins at the high levels at which they were detected, supports the notion, that they play a key role in regulating the levels of commensal microbiota hence preventing bacterial overgrowth that could result in pathology. Interestingly, we also noted high levels of milk proteins such as lactalbumin alpha (LALBA) and alpha & kappa caseins (CSN2 & CSN3). These proteins were present at the highest levels in the first 6 months of life and declined in the second half of the first year of life. Most children over the age of 12 months had relatively low levels of milk proteins in their upper airways. Taken together, these observations are consistent with the fact most infants under the age of six months are predominantly breast-fed and the decline milk-protein levels after six months is most likely associated with weaning. The precipitous decline observed after the first year of life most likely reflects the complete cessation of breast feeding.

Finally, using ordination analysis, we compared the airway proteomes of children with different clinical manifestations of respiratory illness to that of healthy children who were sampled from home. The proteomes of children with respiratory infections clustered separately from those of well children. This distinction is most likely attributable host innate inflammatory responses mounted by the host in order to address an ongoing infection. These responses would be absent in well controls, in whom the absence of any clinical symptoms at the time of sampling, indicates the absence of an intense inflammatory response. Our search of T-cell associated marker and effector proteins, did not identify any such proteins in the upper-airway proteomes of any of the children in this study. T cell effector cytokines and chemokines such as IL-6, IFN-g, IL-10, IL-8 and other mediators have been widely reported in studies of paediatric upper airway secretions using sensitive immunoassays such as luminex and MSD mesoscale. The absence of these mediators in this study, suggest a possible limitation of the methods reported in this paper: the failure to identify very low abundance proteins in the upper airway proteome. In spite of this shortcoming, MS-based methods reported here offer great advantages over targeted approaches by providing an unbiased, hypothesis-free overview of the upper-airway proteome, thereby increasing the likelihood of identifying novel associations with disease pathology.

## Electronic supplementary material

Supplementary information

## Data Availability

Data analysis was done using R. Cluster analysis to determine differences in protein expression between children with respiratory infections and healthy controls was done using nonmetric multidomensional scaling analysis. The accession number for the data reported in this paper is proteomeXchange: PDX009403.
