# Supplementary material for: Untargeted analysis of the airway proteomes of children with respiratory infections using mass spectrometry based proteomics
Source: Sci Rep. 2018 Sep 14;8:13814. doi: 10.1038/s41598-018-32072-3 (PMC6138648; doi:10.1038/s41598-018-32072-3)
Supplement: Supplementary file 1 — Supplementary information [file 41598_2018_32072_MOESM1_ESM.pdf]

Supplementary information

Untargeted analysis of the airway proteomes of children with respiratory infections using mass spectrometry based proteomics

Charles J Sande, Martin Mutunga, Jacqueline Muteti, Jay Berkley, James Nokes, James Njunge

Supplementary methods

Protocol A: Total proteome; Label free quantification (LFQ)

Six randomly selected NPOP swabs that had been collected from infants and children with respiratory illness and stored at -80°C were thawed on ice in 1 ml aliquots and tranfered to 3 kDa amicon filters (Millipore, USA). The filters were centrifuged at 14,000xg at 4°C to facilitate the removal of low molecular weight components of UTM (Salts, L-Cysteine, Gelatin, Sucrose, L-Glutamic Acid, HEPES Buffer, Vancomycin, Amphotericin B, Colistin, Phenol Red) from the samples. After this centrifugation step, the initial volume of 1ml was reduced to 100µl, following which a dialysis buffer of 300µl 8M urea in 50mM ammonium bicarbonate was added to facilitate protein extraction and denaturation. Three successive centrifugation cycles were then conducted using amicon centrifugal filters as above. In each cycle, a further 300µl of dialysis buffer was added to the post-centrifugation retentate and the centrifugation process was repeated. At the end of the three centrifugation cycles, a final protein concentrate of ~100µl was obtained. The proteins were then reduced with 20 mM Dithiothreitol (DTT; Sigma-Aldrich, USA) at room temperature for 1h and subsequently alkylated in the dark for 1 h with 65 mM iodoacetamide (IAA, Sigma-Aldrich, USA). Excess

IAA was quenched using 65 mM DTT and the urea present in the sample was dialyzed out with 50mM ammonium bicarbonate. Samples were transferred to 1.5ml centrifuge tubes for in-solution trypsin digestion. One microgram of sequencing grade Trypsin (Sigma-Aldrich, USA) was added to the protein samples and protein digestion was allowed to proceed overnight at 37°C with shaking. Peptides were desalted using P10 C18 pipette ZipTips (Millipore, USA) according to the manufacturer's instructions. Eluted peptides were dried in a Speedvac concentrator (Thermo Scientific) and re-suspended in 15μl loading solvent (97.05% H_2O, 2% acetonitrile, 0.05% formic acid) awaiting mass spectrometry analysis.

Protocol B: Cellular proteome: LFQ protein quantification

Four randomly selected NPOP samples from infants and children with respiratory infections were retrieved from -80°C and thawed on ice. One-ml aliquots were centrifuged at 14,000 rpm for 10 mins at 4°C to obtain cell pellets. The supernatants were subsequently frozen at -80°C, awaiting further analysis. The cell pellets were washed once using PBS and lysed by bead-vortexing for 10 minutes in cell lysis buffer (RLT, Qiagen, Germany). Proteins (as well as DNA and RNA) were then extracted from the lysate using the AllPrep DNA/RNA/Protein Mini Kit (Qiagen, Germany) following manufacturers instructions. The concentration of total protein obtained was determined using the Bradford assay (Bio-Rad, USA). Thirty micrograms (30μg) of total protein from each sample was then reduced with 10mM tris(2-carboxyethyl)phosphine (TCEP, Sigma-Aldrich, USA) at 55°C for 1h and subsequently alkylated with 18mM IAA (Sigma-Aldrich, USA) for 30 minutes at room temperature, while keeping the reaction protected from light. Proteins were precipitated overnight at -20°C with six volumes of pre-chilled (-20°C) acetone (Sigma-Aldrich, USA). The samples were centrifuged at 8,000xg for 10 minutes at 4°C to obtain the protein pellets and supernatants were discarded. The protein pellet was resuspended in 100μl of 50mM Triethylammonium

bicarbonate (TEAB, Sigma-Aldrich, USA). Trypsin (Sigma-Aldrich, USA) was added to the protein samples at a trypsin-protein sample ratio of 1:10 and protein digestion was allowed to proceed overnight at 37°C with shaking. Peptides obtained were desalted using P10 C18 pipette ZipTips (Millipore, USA) according to the manufacturer's instructions. Eluted peptides were dried in a Speedvac concentrator (Thermo Scientific), and resuspended in 15 µl loading solvent (97.05% H_2O, 2% acetonitrile, 0.05% formic acid) for mass spectrometry analysis.

Protocols $C_{i\,\&\,ii}$: Cellular proteome: Tandem Mass Tagging (TMT) protein quantification

Eighty-three naso-oro-pharyngeal swabs obtained from paediatric patients with respiratory infections were analyzed in protocol C. Protein extraction, and trypsin digestion was carried out as described in Protocol B, above, although 25µg of total protein was used. The peptide samples were randomly assigned to 10 individual batches: the first eight batches contained nine patient samples and one pooled control sample, while batches nine and ten contained five and six patient samples respectively and one pooled control sample each. The pooled control sample consisted of a pool of peptides from all patient samples. The peptide samples derived from individual patients were then individually labelled using the TMT10plex mass tag kit (Thermo scientific, USA) according to manufacturer's instructions, with one isobaric tag being exclusively used to label the pooled control sample. The labelled peptides for each 10plex were subsequently combined to generate 10 individual pools (figure 1) among which the control pooled sample was evenly distributed. The labelled peptide pools were desalted using P10 C18 pipette ZipTips (Millipore, USA) according to the manufacturer's instructions. Eluted peptides were dried in a Speedvac concentrator (Thermo Scientific, USA), and re-suspended in 15 µl loading solvent for mass spectrometry analysis. In protocol C_i, peptides were eluted on a 3h gradient during mass spectrometry analysis while in protocol C_{ii}, the same peptides were eluted on a 6h gradient (see below & figure 1)

Supplementary figure

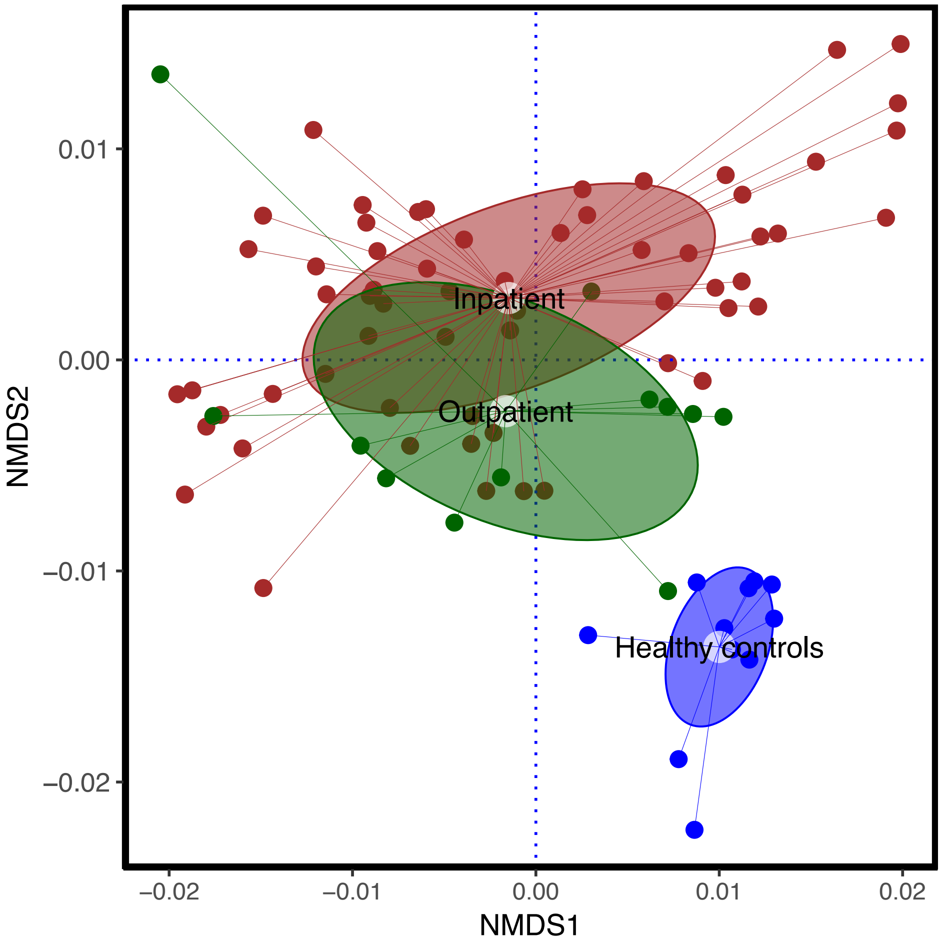

Supplementary figure 1

Supplementary figure legend

Supplementary figure 1

Nonmetric multidimensional scaling (NMDS) was used to visualize the associations between the airway proteomes of children with and without respiratory infections The proteomes of children with respiratory illness clustered together irrespective of whether they were admitted to hospital (inpatients) or not (outpatients). The proteomes of healthy children clustered separately from those of children with respiratory infection.
